# Supplementary material for: Triglyceride-glucose index on risk of adverse events after drug-coated balloon angioplasty
Source: Lipids Health Dis. 2023 Oct 28;22:184. doi: 10.1186/s12944-023-01951-8 (PMC10613374; doi:10.1186/s12944-023-01951-8)
Supplement: Supplementary file 1 — Supplementary Material 1 [file 12944_2023_1951_MOESM1_ESM.pdf]

editage

Editing Certificate

This document certifies that the manuscript listed below has been edited to ensure language and grammar accuracy and is error free in these aspects. The edit was performed by professional editors at Editage, a brand of Cactus Communications. The author's core research ideas were not altered in any way during the editing process. The quality of the edit has been guaranteed, with the assumption that our suggested changes have been accepted and the text has not been further altered without the knowledge of our editors.

MANUSCRIPT TITLE

Triglyceride-glucose index on risk of adverse events after drug-coated balloon angioplasty

AUTHORS

Zhaorong Lin, Xi He, Maosen Lin and Lianglong Chen

ISSUED ON

October 13, 2023

JOB CODE

ULZED_1

THE EDITAGE Promise QUALITY GUARANTEE

Prabh Grewal
Senior Vice President - Editage

editage | helping you get published

Since 2002, Editage has helped over 430,000 authors publish around 1.2 million research papers in scholarly journals across over 1000 disciplines through editorial, translation, transcription, and publication support services. Editage is a brand of Cactus Communications (cactusglobal.com), a science communication and technology company.

CACTUS.

GLOBAL :
+1(833) 979-0061 | request@editage.com

CHINA :
400-120-3020或 021-6020-9400 | fabiao@editage.cn
