# Supplementary material for: Triglyceride-glucose index on risk of adverse events after drug-coated balloon angioplasty
Source: Lipids Health Dis. 2023 Oct 28;22:184. doi: 10.1186/s12944-023-01951-8 (PMC10613374; doi:10.1186/s12944-023-01951-8)
Supplement: Supplementary file 2 — Supplementary Material 2 [file 12944_2023_1951_MOESM2_ESM.pdf]

20231016194532911381434790211584

WORD COUNT	4251	TIME SUBMITTED	16-OCT-2023 07:45PM
		PAPER ID	103510121

Triglyceride-glucose index on risk of adverse events after drug-coated balloon angioplasty

Abstract

Background: The pathogenetic mechanism of atherosclerotic cardiovascular diseases is associated with insulin resistance (IR), which serves as a metabolic risk factor. As an novel indication for IR, triglyceride-glucose (TyG) index may predict cardiovascular disease outcomes.

Methods: In current study, a cohort of 157 individuals with newly developed de novo lesions who received DCB angioplasty between January 2017 and May 2021 were included. The midterm follow-up clinical results consisted of the presence of vessel-oriented composite endpoint (VOCE). The baseline TyG index was divided into three groups by tertiles. This study compared various clinical characteristics and parameters among different groups during DCB angioplasty. A multivariate Cox regression model was built to investigate the potential predictors.

Results: Higher TyG index indicated a increased risk of VOCE according to the adjusted model (HR=4.0, 95%CI: 1.0-15.4, P=0.047). A non-linear correlation was uncovered between the index and VOCE from the smooth curve. Based on Kaplan-Meier curve, individuals in the highest TyG index group was more likely to develop VOCE ($P < 0.05$ for log-rank).

Conclusions: The incidence of VOCE was shown to be independently and positively correlated with an elevated TyG index in individuals with de novo coronary lesions

who received DCB angioplasty.

Keywords: Triglyceride-glucose index; Drug-coated balloons; De novo coronary artery lesions; Vessel-oriented composite endpoint

Background

Drug-coated balloon (DCB) is commonly used in treating various cardiovascular diseases, and cardiologists commonly use the implant-free approach of DCB is widely used by cardiologists which is progressively becoming a standard therapeutic option. By enabling consistent administration of drugs that inhibit cell growth in the inner layer of blood vessels, DCB helps reduce vascular wall thickening and can potentially prevent vascular remodeling by locally dispersing cell-growth-inhibiting drugs [1]. DCBs are effective in treating de novo coronary artery disease, and other conditions [2–5]. The consensus [6] has guided the implementation of the optimal medical intervention. However, individuals who have received drug-coated angioplasty for a similar ailment may develop adverse clinical consequences [7], including vessel-oriented composite endpoint (VOCE). The risk factors and mechanisms of VOCE are complex. Recent studies have indicated that anatomical indexes such as post-procedural percent diameter stenosis (%DS) and post-procedural minimal lumen diameter (MLD) were associated with major adverse cardiovascular events [8, 9]. Identifying risk factors for vessel-focused composite endpoints is essential in developing efficient approaches to decrease the occurrence of VOCE, thus holding significant therapeutic significance.

Insulin resistance (IR) refers to the reduced ability of cells in the body to respond to insulin, which is commonly observed in patients with type 2 diabetes mellitus. This results in the impairment of insulin's ability to regulate glucose metabolism, leading to elevated blood glucose levels and a range of related health complications [10]. Homeostatic Model Assessment (HOMA) for IR and hyperinsulinemia-euglycemic clamp are more complex way to evaluate IR. Additionally, IR currently is the clinically recognized risk cardiometabolic factors for coronary heart disease and can lead to unsatisfactory clinical outcomes [11–13]. As the novel IR indicator, the TyG index can affect the clinical outcomes of individuals with coronary heart disease and other related conditions [14].

By exploring the connections between metabolic markers, coronary pathophysiology, and interventional methods, the study improved the understanding of the TyG index in predicting VOCE. The novel aspect of the IR associated indicator is from the cardiovascular interventional operation application. This study aims to explore whether novel indicator can identify VOCE in patients undergoing DCB angioplasty at an early phase.

Methods

Study design and population

This study included patients with de novo coronary artery lesions who had undergone DCB angioplasty at Fujian Medical University Union Hospital between January 1, 2017, and May 31, 2021. A grand total of 157 individuals were

categorized into three groups by tertiles (Figure 1). The Ethics Committee of Fujian Medical University Union Hospital approved the study protocol and issued the procedure with the approval number 2023KY092. The current study conforms to the Helsinki Declaration.

Inclusion criteria

Patients with available laboratory data, high-quality angiographic images, and de novo coronary artery lesions underwent DCB angioplasty were included.

Exclusion criteria

The present study excluded those with incomplete data on laboratory data such as fasting glucose and fasting triglyceride etc. Additionally, individuals with left main coronary artery disease, ostial lesion, highly calcified or thrombotic lesion, and inadequate angiographic image quality were also excluded.

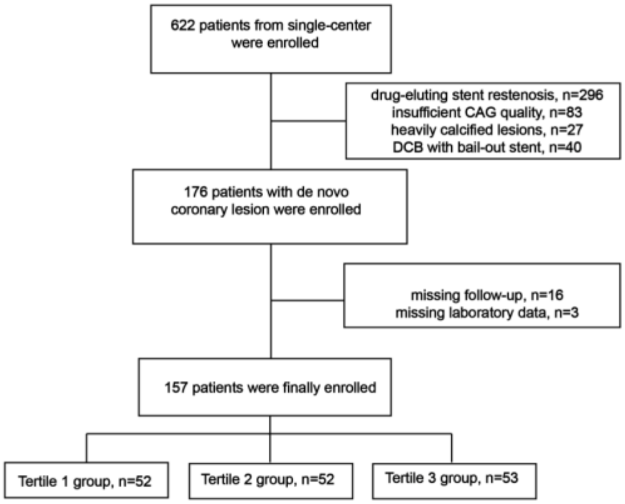

Fig.1 Flow chart of recruitment procedure

Blood Parameter Measurements

Baseline blood samples were collected before the DCB angioplasty to test clinical laboratory indexes. Biochemical detector (Cobas 8000, Roche, Germany) was used to analyze fasting blood samples for plasma lipids, serum creatinine (SCr), and fasting blood glucose (FBG). Glycosylated hemoglobin analyzer (Sysmex G7, Japan) was used to detect hemoglobin A1c (HbA1c), which serves as an indicator of glycemic control level. Furthermore, the formula for calculating TyG index is as follows: .

Statistics

Continuous variables that exhibited an approximately normal distribution were denoted as mean ± standard deviation, when applicable. On the other hand, percentages were used to present for categorical variables. The study used one-way analysis of variance to examine differences in continuous variables across the TyG index tertiles. Sex, hypertension, current smoker, medication and additional categorical variables in this study were examined by Chi-square tests or Fisher's exact tests. The study also employed linear regression analyses to explore the potential relationship among the TyG index and other cardiovascular risk or protection factors, and a correlation heatmap was used to visualize these associations. Continuous variables comparison between the two different groups divided by clinical outcomes entailed the utilization of Student's t-test. To delve into the relationship between potential risk factors and VOCE prevalence, Cox regression

analyses were conducted. Kaplan-Meier curves were used for achieving the survival information of different groups, and the statistical differences among varying levels of the TyG index groups were examined by the log-rank test. Additionally, a smooth curve in present study was employed to strengthen understanding of non-linear relationship between the TyG index and VOCE incidence.

A statistically significant result was determined if the $P < 0.05$. Statistical analyses were conducted using SPSS version 23.0 (SPSS Inc., Chicago, IL, USA), MedCalc version 11.4 (MedCalc Inc., Ostend, Belgium), and R statistical environment version 4.2.2.

Results

296 patients with in-stent restenosis lesions received DCB angioplasty were excluded. Additionally, 83 patients with inadequate and low-quality Coronary angiography (CAG) images, 27 patients with highly calcified lesions, and 40 patients who underwent DCB with bail-out stenting were considered for exclusion. Of the 176 registered participants, 16 were excluded because they missed follow-up information, and three were excluded because they did not have FBG data. For this investigation, a cohort of 157 patients who underwent DCB angioplasty was included, and their follow-up over the midterm was documented.

Table 1 displays detailed baseline information on the patients. The average of the TyG index in different groups were 8.2 ± 0.2, 8.7 ± 0.1, and 9.4 ± 0.4, correspondingly. The average age of the three distinct tiers of the TyG index groups

were 64.1 ± 10.3, 58.8 ± 12.3, and 62.0 ± 10.6, correspondingly.The TyG index's lowest tertiles exhibited the greatest level of high-density lipoprotein cholesterol (HDL-C) (1.1 ± 0.3 compared to 1.0 ± 0.3 and 1.0 ± 0.3, $P<0.001$). Tertile 3 had the highest TG levels, FBG levels, HbA1c levels, incidence of diabetes mellitus, and beta-blocker usage ($P<0.05$). Three groups demonstrated no notable variations in age, body mass index (BMI), clinical presentation, laboratory findings, medication, lesion characteristics, gender, and DCB angioplasty parameters.

Table 1. Baseline laboratory indicators, medication, vessel and DCB characteristics by tertiles of the TyG index

Variable	Tertile 1 (n=52)	Tertile 2 (n=52)	Tertile 3 (n=53)	*P* value
TyG index	8.2 ± 0.2	8.7 ± 0.1	9.4 ± 0.4	<0.001
Age,years	64.1 ± 10.3	58.8 ± 12.3	62.0 ± 10.6	0.056
Body mass index,kg/m2	23.8 ± 2.9	24.9 ± 3.2	25.0 ± 3.0	0.059
LDL-C,mmol/L	2.7 ± 1.0	2.7 ± 1.0	2.9 ± 1.3	0.547
TC,mmol/L	4.0 ± 1.1	4.1 ± 1.1	4.4 ± 1.4	0.143
HDL-C,mmol/L	1.1 ± 0.3	1.0 ± 0.3	1.0 ± 0.3	<0.001
SCr,umol/L	75.6 ± 21.8	91.4 ± 85.7	79.1 ± 20.6	0.272
LVEF,%	63.0 ± 9.8	63.8 ± 8.9	63.3 ± 10.3	0.916
triglyceride,mg/dL	83.3 ± 18.7	131.1 ± 23.7	206.5 ± 91.0	<0.001
Fasting blood glucose,mg/dL	94.4 ± 17.0	99.0 ± 15.5	134.6 ± 45.8	<0.001
Hemoglobin A1C,%	6.5 ± 1.1	6.6 ± 1.5	7.3 ± 1.6	0.007
Reference vessel diameter,mm	2.5 ± 0.7	2.6 ± 0.8	2.5 ± 0.6	0.521
Maximum drug-coated balloon diameter,mm	2.6 ± 0.4	2.7 ± 0.4	2.6 ± 0.5	0.798
Maximum drug-coated pressure, atm	10.8 ± 2.0	10.3 ± 2.4	10.8 ± 3.0	0.532
Men,n(%)	39 (75.0%)	41 (78.8%)	42 (79.2%)	0.847
ACS,n(%)	43 (82.7%)	44 (84.6%)	45 (84.9%)	0.945
Hypertension,n(%)	33 (63.5%)	34 (65.4%)	36 (67.9%)	0.89
Diabetes mellitus,n(%)	16 (30.8%)	17 (32.7%)	33 (62.3%)	0.001

Current smoker,n(%)	19 (36.5%)	14 (26.9%)	14 (26.4%)	0.445
ACEI/ARB/ARNI,n(%)	29 (55.8%)	31 (59.6%)	30 (56.6%)	0.917
Beta-blocker,n(%)	34 (65.4%)	30 (57.7%)	47 (88.7%)	0.001
Statins,n(%)	52 (100.0%)	52 (100.0%)	52 (98.1%)	0.373
Ticagrelor,n(%)	36 (69.2%)	36 (69.2%)	44 (83.0%)	0.177
Clopidogrel,n(%)	15 (28.8%)	16 (30.8%)	9 (17.0%)	0.213
Aspirin,n(%)	50 (96.2%)	50 (96.2%)	50 (94.3%)	0.873
Type of predilation drug				0.419
Cutting balloon,n(%)	21 (40.4%)	13 (25.0%)	15 (28.3%)	
Semicompliant balloon,n(%)	23 (44.2%)	23 (44.2%)	27 (50.9%)	
Noncompliant balloon,n(%)	2 (3.8%)	5 (9.6%)	2 (3.8%)	
Both/All,n(%)	6 (11.5%)	11 (21.2%)	9 (17.0%)	
Type of target vessel				0.198
LAD,n(%)	25 (48.1%)	24 (46.2%)	25 (47.2%)	
LCX,n(%)	17 (32.7%)	16 (30.8%)	10 (18.9%)	
RCA,n(%)	5 (9.6%)	11 (21.2%)	12 (22.6%)	
Branch,,n(%)	5 (9.6%)	1 (1.9%)	6 (11.3%)	
Post-procedure TIMI grade 3 flow,n(%)	51 (98.1%)	51 (98.1%)	52 (98.1%)	1.000
Drug coated balloon diameter to vessel diameter ratio(1:1),n(%)	12 (23.1%)	12 (23.1%)	12 (22.6%)	0.998

The data is presented as either a number (%) or as the mean ± standard deviation (SD). ACEI refers to angiotensin-converting enzyme inhibitor, while ARB stands for angiotensin II receptor blocker. SCr represents serum creatinine, LVEF represents left ventricular ejection fraction, ACS represents acute coronary syndrome, LAD represents left anterior descending artery, LCX represents left circumflex artery, LDL-C represents low-density lipoprotein cholesterol, RCA represents right coronary artery, RLD represents reference lumen diameter, TC represents total cholesterol, TIMI stands for thrombolysis in myocardial infarction, and TyG index refers to triglyceride-glucose index.

Table 2 demonstrates that the TyG index levels positively correlated with BMI, HbA1c, and low-density lipoprotein cholesterol (LDL-C) while displaying negative correlation with HDL-C ($P < 0.05$). The correlation heatmap in Figure 2 displayed

the connection among the cardiovascular laboratory variables. Throughout the midterm follow-up, the VOCE occurrences were documented, comprising of one (0.6%) cardiac death related to vessels, four (2.5%) myocardial infarctions related to vessels, and 13 (8.3%) revascularizations driven by ischemia in the target vessel. The analysis for VOCE using Cox regression is displayed in Table 3. Elevated TyG index values are associated with a higher risk of VOCE (Tertile 3 group HR 4.0, 95% CI 1.0 , 15.4, *P*=0.047). A noteworthy point is that the predictive capability remained even after adjusting for LVEF, age, Scr, HbA1C, LDL, hypertension, acute coronary syndrome, HDL, BMI, sex, current smoke status, and DM (*P*=0.022 for the trend).

Table 2. Analysis of TyG index and cardiovascular associated factors using both univariate and multivariate linear regression

Variable	Univariate		Multivariate	
	β(95% CI)	*P* value	β(95% CI)	*P* value
HbA1c	0.1110 (0.0535, 0.1684)	<0.001	0.1168 (0.0645, 0.1691)	<0.001
BMI	0.0413 (0.0134, 0.0692)	0.004	0.0398 (0.0147, 0.0649)	0.002
HDL-C	-0.5769 (-0.8829, -0.2708)	<0.001	-0.6715 (-0.9702, -0.3728)	<0.001
Scr	0.0005 (-0.0012, 0.0021)	0.583	0.0002 (-0.0014, 0.0017)	0.845
LDL-C	0.0697 (-0.0078, 0.1471)	0.080	0.1171 (0.0461, 0.1881)	0.001

Abbreviations see Table 1

Table 3. Baseline TyG index and prediction of VOCE

TyG index groups	Non-adjusted model		Full-adjusted model	
	HR (95% CI)	*P* value	HR (95% CI)	*P* value
T1	Ref		Ref	
T2	0.6 (0.1, 2.5)	0.481	0.9 (0.2, 4.5)	0.92
T3	2.0 (0.7, 6.0)	0.198	4.0 (1.0, 15.4)	0.047
P for-trend		0.124		0.022

The variables that were modified in full-adujsted model included LVEF, age, serum creatinine,

sex, HbA1c, low-density lipoprotein, hypertension, acute coronary syndrome, high-density lipoprotein, body mass index, current smoking status, diabetes mellitus.

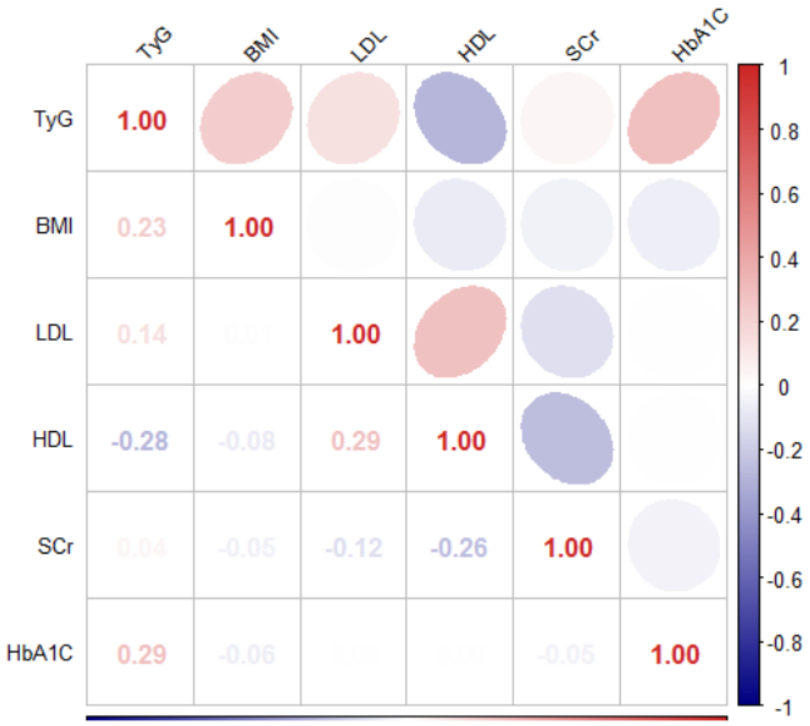

Figure 2. Correlation heatmap among cardiovascular laboratory factors

After analyzing their clinical outcomes using CAG images during follow-up, the present study categorized them into two groups: the VOCE group consisting of 18 patients and the non-VOCE group consisting of 139 patients. In the non-VOCE group, the median follow-up time was 345 days, while in the VOCE group it was 278 days ($P > 0.05$). Table 4 displays the features of the lesions and procedures. Out of the 157 vessels assessed, 64 (46.0%) in the non-VOCE group and 10 (55.6%) in the VOCE group were identified as left anterior descending arteries. Additionally, 38 (27.3%) in the non-VOCE group and five (27.8%) in the VOCE group were associated with left

circumflex arteries. Moreover, 25 (18.0%) in the non-VOCE group and three (16.7%) in the VOCE group were linked to right coronary arteries. Lastly, 12 (8.6%) in the non-VOCE group were identified to be branches of coronary arteries. No notable variances were observed in the categories of predilation balloons and the proportion of DCB diameter to vessel diameter 1:1, post-procedural thrombolysis in myocardial infarction (TIMI) grade 3 flow, maximum DCB diameter, maximum DCB pressure, and reference vessel diameter (RVD). Table 4 displays that individuals in different groups were similar to age, gender, BMI, clinical symptoms, laboratory results, and medication, except for a higher prevalence of hypertension (69.1% vs. 38.9%, P=0.011) in the non-VOCE group compared to the VOCE group. Table 5 presents the factors that exhibited a notable connection with an elevated risk of VOCE. These factors include the TyG index (HR 2.3, 95%CI 1.0–5.1, P=0.047). Upon accounting for covariates, the multivariable analysis demonstrated a continued and substantial correlation between the TyG index (HR 4.0, 95% CI 1.1–14.7, P=0.035) and an escalated risk of VOCE.

Table 4. Clinical and lesion characteristics

Variables	Non-VOCE(n=139)	VOCE(n=18)	*P*-value
Age,years	62 ± 11	59 ± 13	0.207
Body mass index,kg/m^2	24.7 ± 3.1	23.6 ± 2.6	0.155
LDL-C,mmol/L	2.7 ± 1.1	3.0 ± 1.4	0.34
TC,mmol/L	4.1 ± 1.2	4.5 ± 1.4	0.225
HDL-C,mmol/L	1.0 ± 0.3	1.1 ± 0.3	0.514
SCr,umol/L	83.2 ± 55.5	73.2 ± 9.6	0.448

LVEF,%	63.4 ± 9.5	63.3 ± 11.2	0.965
Triglyceride,mg/L	138.3 ± 74.7	159.9 ± 79.0	0.253
Triglyceride-glucose index	8.8 ± 0.5	9.0 ± 0.6	0.058
Fasting blood glucose,mg/dL	107.7 ± 34.0	123.3 ± 37.6	0.072
Hemoglobin A1C,%	6.8 ± 1.5	7.0 ± 1.4	0.485
Reference vessel diameter,mm	2.6 ± 0.7	2.4 ± 0.6	0.371
Maximum drug-coated balloon diameter,mm	2.6 ± 0.5	2.7 ± 0.5	0.747
Maximum drug-coated pressure, atm	10.6 ± 2.6	10.6 ± 2.1	0.972
Men,n(%)	106 (76.3%)	16 (88.9%)	0.226
ACS,n(%)	117 (84.2%)	15 (83.3%)	0.927
Hypertension,n(%)	96 (69.1%)	7 (38.9%)	0.011
Diabetes mellitus,n(%)	57 (41.0%)	9 (50.0%)	0.467
Current smoker,n(%)	39 (28.1%)	8 (44.4%)	0.153
ACEI/ARB/ARNI,n(%)	81 (58.3%)	9 (50.0%)	0.504
Beta-blocker,n(%)	97 (69.8%)	14 (77.8%)	0.483
Statins,n(%)	138 (99.3%)	18 (100.0%)	0.718
Ticagrelor,n(%)	105 (75.5%)	11 (61.1%)	0.19
Clopidogrel,n(%)	33 (23.7%)	7 (38.9%)	0.165
Aspirin,n(%)	132 (95.0%)	18 (100.0%)	0.33
Type of predilation drug			0.538
Cutting balloon,n(%)	42 (30.2%)	7 (38.9%)	
Semicompliant balloon,n(%)	67 (48.2%)	6 (33.3%)	

Noncompliant balloon,n(%)	7 (5.0%)	2 (11.1%)	
Both/All,n(%)	23 (16.5%)	3 (16.7%)	
Type of target vessel			0.598
LAD,n(%)	64 (46.0%)	10 (55.6%)	
LCX,n(%)	38 (27.3%)	5 (27.8%)	
RCA,n(%)	25 (18.0%)	3 (16.7%)	
Branch,,n(%)	12 (8.6%)	0 (0.0%)	
Post-procedure TIMI grade 3 flow,n(%)	137 (98.6%)	17 (94.4%)	0.23
Drug-coated balloon diameter to vessel diameter ratio(1:1),n(%)	137 (98.6%)	17 (94.4%)	0.23

Abbreviations see Table 1

Table 5. Associated factors with VOCE

Variables	Univariate analysis		Multivariate analysis	
	HR(95%CI)	*P*-Value	HR(95%CI)	*P*-Value
Triglyceride-glucose index	2.3 (1.0, 5.1)	0.047	4.0 (1.1, 14.7)	0.035
Maximum drug-coated pressure, atm	0.9 (0.8, 1.1)	0.489		
Post-procedure TIMI grade 3 flow,n(%)	0.3 (0.0, 2.5)	0.274		
Maximum drug-coated balloon diameter,mm	1.2 (0.5, 3.3)	0.651		
ACS	0.9 (0.3, 3.2)	0.899		
Hemoglobin A1C	1.1 (0.8, 1.4)	0.586		
LVEF	1.0 (1.0, 1.1)	0.85		
SCr	1.0 (1.0, 1.0)	0.322		
HDL-C	2.8 (0.5, 16.2)	0.24		

Hypertension	0.4 (0.1, 1.0)	0.044
LDL-C	1.3 (0.9, 1.9)	0.172
TC	1.3 (1.0, 1.9)	0.089
Diabetes mellitus	1.3 (0.5, 3.2)	0.156
BMI	0.9 (0.8, 1.0)	0.156
Current smoker	2.0 (0.8, 5.2)	0.133

Abbreviations see Table 1

Based on the assessment of the Kaplan–Meier curve presented in figure 3, the cohort with a TyG index exceeding 8.95 exhibited inferior outcomes throughout the mid-term follow-up phase. Utilizing the outcomes of the tertile 2 group as a baseline, the log-rank test demonstrated a noteworthy disparity between the tertile 2 and tertile 3 groups ($P = 0.037$). The application of a smooth curve fitting method to investigate the positive correlation between the TyG index and VOCE is illustrated by Figure. 4.

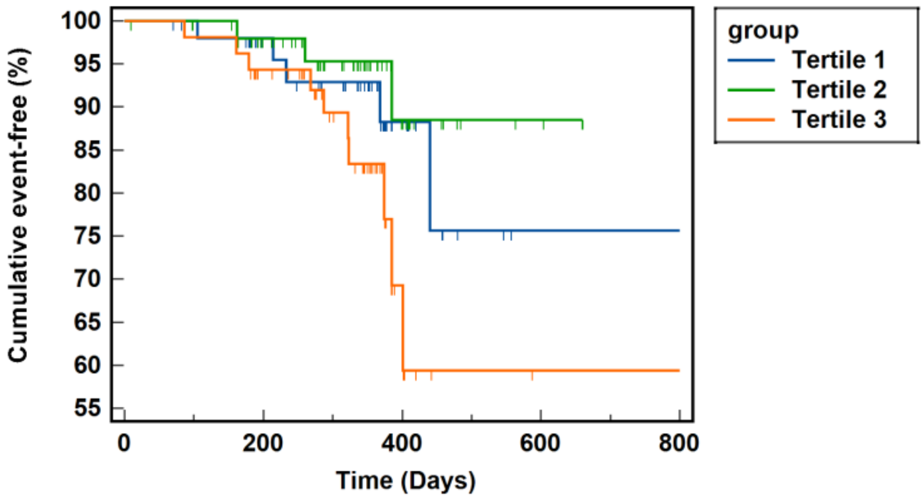

Figure 3. Patients who underwent drug-coated balloon angioplasty were analyzed using Kaplan-Meier curves.

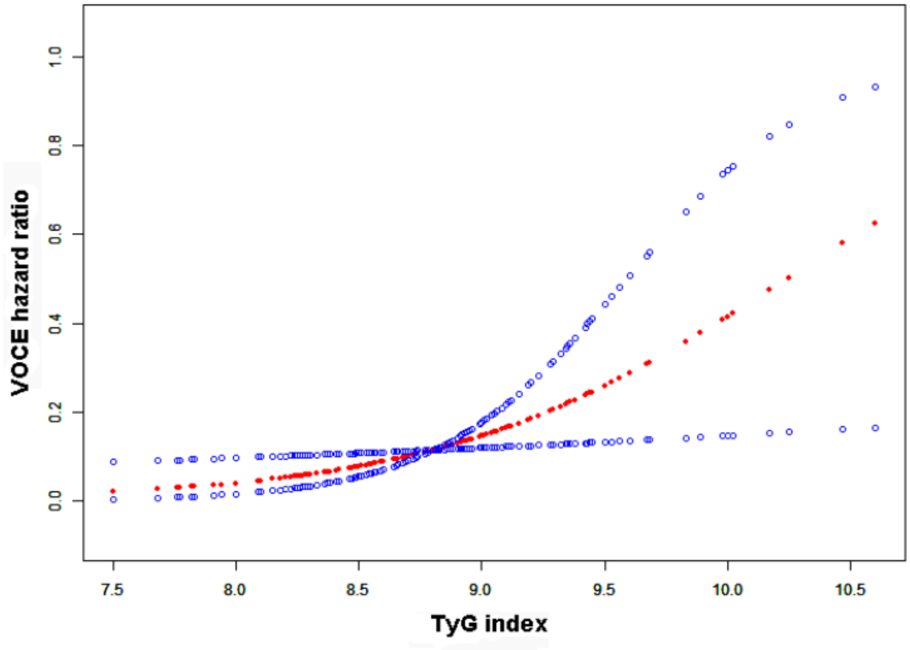

Figure 4. The correlation between the TyG index and VOCE

The smooth curve fitting between variables is represented by a solid line in the middle. The dashed line on either side indicates a 95% CI for the fit. The TyG index displays the population density through a black and white strip at the bottom.Model was adjusted for LVEF, age, Scr, sex, HbA1C, LDL, hypertension, ACS, HDL, BMI, current smoke status and DM.

Discussion

Based on the present understanding, this study is the first investigation that focused on the relationship between clinical outcomes and the novel index in patients with de novo coronary lesions who received DCB angioplasty. The primary outcomes of this investigation were as follows: (1) The TyG indicator exhibited a correlation with cardiovascular laboratory variables. (2) Higher TyG index levels were associated with unfavorable cardiovascular clinical outcomes during the midterm follow-up

period. (3) Increased TyG index levels, whether measured continuously or categorically, were found to be independently linked to a heightened risk of VOCE in the fully adjusted model, accounting for confounding factors.

Inflammation is a significant cause of a variety of heart conditions [15, 16]. The interaction of genetic, environmental, and behavioral influences induces IR. The reduced sensitivity and responsiveness of insulin affects the metabolism of fats and sugar, potentially worsening high blood glucose levels and abnormal lipid levels. An association has been found between high HbA1c levels and cardiovascular risk, according to a randomized clinical trial [17]. The association of TyG index and VOCE incidence being independent of HbA1C suggests that TyG index may provide additional information regarding cardiovascular risk factors, such as atherogenic lipoprotein, which is beyond traditional glucose control indicator. TG and parts of cholesterol are transported in the circulation by TG-rich lipoproteins (TRL) that contain chylomicrons and very low-density lipoprotein (VLDL). The development of atherosclerotic injury is triggered and exacerbated by atherogenic lipoproteins, specifically LDL, VLDL, and TRL [18, 19]. The aforementioned lipoproteins promote the accumulation of lipids, formation of foam cells, inflammatory responses, and dysfunction of the endothelial cells. Researches indicates that patients with chronic diseases like prediabetes may face a higher cardiovascular risk due to elevated plasma levels of TRL [20, 21]. TRL plays a pivotal role in the progression of atherosclerosis and should be an indispensable focus for both therapeutic intervention and research aimed at reducing cardiovascular risk in the future. Several previous

metabolism associated studies demonstrated the index was a more reliable and superior IR biomarker [22, 23]. The worldwide incidence of cardiovascular diseases linked to IR is on the rise, primarily attributed to vascular stiffness, a recognized risk factor for coronary arteriosclerosis [24]. Previous research indicated that the TyG index could positively correlated with arterial rigidity, plaque formation in the carotid artery, damage to the microvessels in the kidneys, and diseases affecting the brain [25, 26]. Microvascular or macrovascular injury may be observed in patients after DCB angioplasty. The mechanism of vascular stiffness may be associated with IR which depresses the production of nitric oxide (NO). The progression of coronary artery disease in individuals with long-term survival periods may be closely associated with inflammation and vascular cell migration caused by NO [27]. Ion theory displays that the amiloride-sensitive channel known as the endothelial cell Na+ channel is crucial in vascular stiffness. Emerging evidence demonstrates that activated endothelium Na+ channel (EnNaC) contributed to cardiovascular stiffening, while activated endothelial cell mineralocorticoid receptor (ECMR) may induce endothelial cell stiffness by EnNaC [27-29]. Based on ion theory, the mechanism of vascular stiffness was dependent on increased ECMR and EnNaC.

It remains unclear how the TyG index affects cardiovascular clinical outcomes. Within the CUN cohort study, which centered around the vascular metabolism of 5014 healthy Caucasian individuals, a significant association was observed. A recent study demonstrated the novel IR index was positively associated with subsequent cardiovascular events [30]. As the TyG index becomes an increasingly important

factor in assessing the risk, the index can aid in making informed clinical decisions and developing preventive strategies. Atherogenic lipoproteins are significantly reduced by proprotein convertase subtilisin/kexin type 9 inhibitors (PCSK9-i), leading to a lower incidence of myocardial infarction and stroke. Long-term mortality risk following acute coronary syndrome may be reduced with PCSK9-i when combined with intensive statin therapy [31]. Moreover, PCSK9-i has effectively reduced atherogenic risk in patients with familial hypercholesterolemia [32]. This study highlights the significant role of PCSK9-i in lipid-lowering therapy for high-risk cardiovascular patients, which can enhance and optimize individual lipid management treatments. It is crucial to use PCSK9-i as a component of a more comprehensive cardiovascular risk management strategy. Prospective studies can be conducted to investigate if combining PCSK9-i treatment with DCB angioplasty can decrease the occurrence of VOCE. The TyG index in Koreans without diabetes mellitus exhibited greater predictive ability for ischemic heart disease than FBG or TG [33][1]. The TyG index demonstrated a noteworthy influence on the progression of cardiovascular disease, cardiovascular mortality, and myocardial infarction, particularly within low-income countries. This was discovered in the Prospective Urban Rural Epidemiology study, which included 141,243 participants from five continents [34]. Further research has indicated an elevated TyG index may provide an innovative indicator of coronary artery disease symptomatology [35, 36]. The TyG index in a longitudinal study conducted in Korea has the potential to independently predict coronary artery calcification [37]. Additionally, when the TyG index is

included in the risk model for major adverse cardiac events in diabetic individuals with ACS, its predictive power is enhanced [38]. Anatomical measurements such as MLD and %DS are commonly employed in intervention to evaluate the extent of narrowing in the blood vessel using CAG. VOCE was identified to be correlated with the degree of vessel diameter stenosis observed on the angiography [39]. Hidekuni Kirigaya et al. discovered that the utilization of post-procedural %DS could potentially aid in making clinical judgments and enhance the overall clinical results for individuals undergoing balloon angioplasty [8]. During DCB angioplasty, individuals with de novo coronary lesions may benefit from the TyG index as a new prognostic factor. Additional research is required to validate if incorporating the TyG index with post-procedural %DS or other anatomical variables into an existing risk prediction model could enhance the capacity to identify patients susceptible to VOCE. Patients diagnosed with coronary artery disease who were admitted for coronary angiography and subsequently received DCB angioplasty have significantly increased as a result of advancements in non-implant intervention technology. Identifying and anticipating the risk of VOCE in conjunction with established risk factors is crucial. Before DCB angioplasty, cardiologists can potentially spot individuals at an elevated risk of developing certain conditions by monitoring fluctuations in their TyG index, and implementing preventative measures to delay or prevent their onset. Further research is needed to fully understand its potential in predicting long-term outcomes and identifying patients at risk of metabolic complications.

Study strengths and limitations

This study efficiently investigates how the TyG index affects the clinical outcomes of patients who underwent DCB. This computational model for calculating the TyG index considers the levels of glucose and lipids in cardiovascular health. TG and glucose levels are frequent laboratory indicators that can be measured from blood samples obtained before the operation. The current study could potentially offer valuable insights into the cardiovascular intervention operation treatment through optimazed clinical management of the TyG index. All analyses were adjusted for important confounders. Regression analyses were conducted based on tertiles and continuous values of the TyG index.

22

The study has certain limitations. First, the retrospective single-center research has a small sample of individuals from the Chinese population. Consequently, the outcomes of the present investigation may not apply to other populations. Second, the TyG index was exclusively obtained from the study sample, necessitating further investigation to validate the threshold value. Third, this study only used the initial TyG index to explore its predictive value. Moreover, there was a lack of substantial proof regarding the combined impact of %DS, MLD, quantitative flow ratio, and the TyG index. Therefore, testing whether the coexistence of vessel anatomical or physiological parameters and the TyG index would increase cardiovascular risk is worthwhile. Fourth, this study solely focused on de novo coronary lesions, and the limited sample size prevented any investigation into other intricate forms of coronary lesions. Fifth, this study was not a preplanned analysis. The potential biases and

confounding factors that were not adjusted for and could have influenced this observational analysis. Hence, future validation of the findings necessitates extensive, meticulously planned, and wide-ranging prospective multicenter investigations.

Conclusions

Patients with a higher TyG index who received DCB angioplasty had a higher risk of VOCE. The index could be widely used in disease management to assist individuals in reducing the risk of developing VOCE after interventional operation, providing a convenient and cost-effective approach.

20231016194532911381434790211584

ORIGINALITY REPORT

9%

SIMILARITY INDEX

PRIMARY SOURCES

1	"Abstracts", European Heart Journal, 1997.<br>Crossref	34 words — 1%
2	cardiab.biomedcentral.com<br>Internet	28 words — 1%
3	www.researchgate.net<br>Internet	26 words — < 1%
4	Takuya Hashimoto, Yoshiyasu Minami, Kiyoshi Asakura, Masahiro Katamine et al. "Lower levels of low-density lipoprotein cholesterol are associated with a lower prevalence of thin-cap fibroatheroma in statin-treated patients with coronary artery disease", Journal of Clinical Lipidology, 2021<br>Crossref	22 words — < 1%
5	"Full Issue PDF", Journal of the American College of Cardiology, 2019<br>Crossref	20 words — < 1%
6	academic.oup.com<br>Internet	20 words — < 1%
7	www.liebertpub.com<br>Internet	20 words — < 1%
8	iovs.arvojournals.org<br>Internet	

19 words — < 1%

9 www.arab-board.org
Internet
18 words — < 1%

10 www.mdpi.com
Internet
18 words — < 1%

11 Konstadina Darsaklis, Matthew E. Dickson, William Cornwell, Colby R. Ayers, Fernando Torres, Kelly M. Chin, Susan Matulevicius. "Right atrial emptying fraction non-invasively predicts mortality in pulmonary hypertension", The International Journal of Cardiovascular Imaging, 2016
Crossref
15 words — < 1%

12 ijph.tums.ac.ir
Internet
15 words — < 1%

13 rcastoragev2.blob.core.windows.net
Internet
15 words — < 1%

14 "Scientific Abstracts", Journal of General Internal Medicine, 2009
Crossref
14 words — < 1%

15 www.imrpress.com
Internet
14 words — < 1%

16 www.oncotarget.com
Internet
13 words — < 1%

17 Cuisset, T.. "Benefit of a 600-mg Loading Dose of Clopidogrel on Platelet Reactivity and Clinical Outcomes in Patients With Non-ST-Segment Elevation Acute
12 words — < 1%

Coronary Syndrome Undergoing Coronary Stenting", Journal of the American College of Cardiology, 20061003
Crossref

18 www.ncbi.nlm.nih.gov
Internet
12 words — < 1%

19 digital.library.adelaide.edu.au
Internet
11 words — < 1%

20 hal.science
Internet
11 words — < 1%

21 pure.rug.nl
Internet
10 words — < 1%

22 Hirotaka Momose, Makoto Takahashi, Masaya Kawai, Kiichi Sugimoto et al. "Risk factors for anastomotic leakage in Low Anterior Resection with a Diverting stoma for rectal cancer: A case control study", Research Square Platform LLC, 2023
Crossref Posted Content
9 words — < 1%

23 J. Hausleiter. "Prevention of restenosis by a novel drug-eluting stent system with a dose-adjustable, polymer-free, on-site stent coating", European Heart Journal, 05/25/2005
Crossref
9 words — < 1%

24 Volpe, Cristina. "Primary Aldosteronism Studies on Screening, Outcome of Adrenalectomy and Functional Pathology", Karolinska Institutet (Sweden), 2021
ProQuest
9 words — < 1%

25 docplayer.net
Internet
9 words — < 1%

arno.unimaas.nl

26	Internet	8 words — < 1%
27	etda.libraries.psu.edu<br>Internet	8 words — < 1%
28	recintervcardiol.org<br>Internet	8 words — < 1%
29	wjso.biomedcentral.com<br>Internet	8 words — < 1%
30	www.degruyter.com<br>Internet	8 words — < 1%
31	www.e-dmj.org<br>Internet	8 words — < 1%
32	www.frontiersin.org<br>Internet	8 words — < 1%
33	www.researchsquare.com<br>Internet	8 words — < 1%

EXCLUDE QUOTES	OFF	EXCLUDE SOURCES	OFF
EXCLUDE BIBLIOGRAPHY	ON	EXCLUDE MATCHES	OFF
